# Patient expectation fulfilment following total hip arthroplasty: a 10-year follow-up study

**DOI:** 10.1007/s00402-020-03430-6

**Published:** 2020-04-01

**Authors:** Liam Z. Yapp, Nicholas D. Clement, Deborah J. Macdonald, Colin R. Howie, Chloe E. H. Scott

**Affiliations:** 1 Department of Trauma and Orthopaedics, Royal Infirmary of Edinburgh, 51 Little France Crescent, Edinburgh, EH16 4SY Scotland, UK; 2 Department of Orthopaedics, University of Edinburgh, Edinburgh, Scotland, UK

**Keywords:** Total hip arthroplasty, Expectations, Fulfilment, Outcome

## Abstract

**Background:**

The primary aim of this study was to assess how expectation fulfilment changes up to 10 years following total hip arthroplasty (THA).

**Materials and methods:**

Three hundred and forty-six patients completed an expectation questionnaire (encompassing 18 activities), Oxford hip score (OHS) and Short Form (SF)-12 prior to surgery. At 1 year postoperatively, expectation fulfilment was assessed in addition to OHS, SF-12 and patient satisfaction (*n* = 346). This was repeated in surviving patients with intact THAs at 9.1–9.9 years postoperative (*n* = 224). Linear regression analysis was used to identify factors independently associated with early (1 year) and late (mean 9.5 years) expectation fulfilment.

**Results:**

Postoperative expectation fulfilment scores declined from 36.5 at 1 year to 33 at late follow-up (95% confidence intervals (CI) 0.0–5.0, *p* < 0.001). Increased (better) late expectation fulfilment scores were significantly associated with better scores for all PROMs applied at both timepoints. Younger age, greater pre-operative expectation score and greater improvement in OHS (both early and late) were all independent predictors when adjusting for confounding (*p* < 0.05). At late follow-up 78% (14/18) activities demonstrated high levels of persistent expectation fulfilment. Approximately two out of every five patients who considered themselves unfulfilled at early follow-up went on to experience late fulfilment, but this was dependent upon the specific expectation (mean 40%, range 0–64%).

**Conclusions:**

Expectation fulfilment following THA changes with time. The majority of patients report high levels of expectation fulfilment following THA at late follow-up. This information can be used to help manage the longer-term expectations of patients undergoing THA.

## Introduction

Total hip arthroplasty (THA) for end-stage osteoarthritis (OA) is effective in reducing hip pain, improving function, enhancing quality of life and is a cost-effective intervention [1–4]. As a result, it is associated with high levels of patient satisfaction [5].

However, a proportion of patients will consider themselves dissatisfied following THA [5]. The causes for this are complex and multi-factorial. For TJA to be considered successful it must provide pain relief, functional recovery and satisfaction without complications [6]. Although, pre-operative hip specific patient reported outcome measures (PROMs) have not demonstrated predictive accuracy in relation to post-operative patient satisfaction [7], a post-operative Oxford Hip scores (OHS) greater than 38 at 12 months has been associated with post-THA satisfaction [3].

Pre-operative expectations likely motivate patient desire for THA surgery and subsequent expectation fulfilment may be considered a marker of treatment success [8]. However, the current literature demonstrates conflicting evidence regarding the role of pre-operative expectations and subsequent levels of satisfaction [8–10]. Several studies have demonstrated a link between post-operative expectation fulfilment at 1 year and treatment success when measured by satisfaction and validated PROMS [8, 10]. Several patient-specific factors have been identified as potential predictors of expectation fulfilment such as age, gender, depression, social deprivation, co-morbid health status and pre-operative function [8, 10–13].

Overall satisfaction may be high following THA, but previous studies have reported that medium to long-term quality of life may be impaired, compared with the normal age-matched population [14]. Consequently, it is unclear whether temporal variation exists in the degree of expectation fulfilment following THA and whether this impacts upon the long-term satisfaction and quality of life derived from this procedure. As pooled analyses of nationally collected registry data have demonstrated that THA patients can expect implant survival between 70 and 85% at 20 years [15], there is a need to understand how patient’s expectation fulfilment evolves to adequately counsel patients.

The primary aim of this study was to assess whether expectation fulfilment changes over the course of 10 years following THA. The secondary aim was to define whether specific expectations that were considered unmet at 1 year, ultimately became fulfilled in the longer term.

## Patients and methods

Ethical approval was obtained for this prospective cohort study. During the study period (January 2009 to June 2010), 395 consecutive primary THAs were undertaken at a university-affiliated teaching hospital. Each procedure was either performed or supervised by one of thirteen consultant orthopaedic surgeons. All patients underwent a standardised THA rehabilitation programme and all data was collected prospectively. Patients were excluded from this study if they had incomplete 1-year PROMs data, as it would not be possible to determine how their expectations had evolved over time. Subjects who had undergone bilateral procedures or underwent revision of their original THA during the period of follow-up were also excluded as it was felt that this would skew the patient’s degree of expectation fulfilment and would not be directly comparable to those whose primary THA was intact. Finally, patients who refused to contribute, no longer retained capacity to participate in the study or were uncontactable were considered lost to follow-up.

Demographic data, depression and pain in other joints and levels of social deprivation were recorded prior to surgery. Social deprivation was determined using the Scottish Index of Multiple Deprivation (SIMD) [16]. This a system created by the Scottish government which splits the country into 6976 ‘data zones’. This enables areas of the population to be assigned a deprivation quintile according to post code based upon indicators of deprivation such as employment, income, crime, housing, health, education and access to services. Using this system, Quintile 1 includes the 20% most deprived data zones and Quintile 5 includes the 20% least deprived data zones.

In addition, all patients completed a questionnaire including the short form (SF-) 12 general health questionnaire [17], the Oxford Hip Score (OHS) [18] and the Hospital of Special Surgery (HSS) Hip Surgery Expectations Survey [8, 10]. The SF-12 is a validated questionnaire which is comprised of physical and mental component summary (PCS and MCS, respectively) scores [17]. The PCS and MCS are calculated using the scores of twelve questions and range from 0–100 (zero represents the lowest level of health and 100 indicates the highest level of health, respectively). The OHS is a validated hip score with 12 questions, each with five possible answers. The scores could range from 0–48, and a higher score signifies better function [18].

The HSS Hip Surgery Expectations Survey is a validated PROM examining expectations of specific hip joint related activities [8]. Patients indicated their expectation level of 18 hip activities on a 5-point Likert scale: ‘very important’; ‘somewhat important’; ‘a little important’; ‘I do not expect this’; or ‘this does not apply to me’. Completed questionnaires were collected at a nurse-led pre-assessment clinic, where they received a standardized information booklet describing the procedure, the intended benefits, the associated complications, rehabilitation and expected outcome.

Each patient received a follow-up questionnaire including the HSS hip expectation fulfilment, patient satisfaction, OHS and SF-12 at 1 year postoperatively and again in November 2018 (considered early and late follow-up, respectively). Satisfaction was measured at both post-operative time-points (“How satisfied are you with your operated hip?”) using a 5-point Likert scale ranging from 'Very dissatisfied’ to ‘Very satisfied’. Patients indicated expectation fulfilment on a 5-point Likert scale with individual expectations fulfilled: ‘greatly’; ‘a lot’; ‘a little’; ‘I did not expect this’; or ‘this did not apply to me’. Those who did not reply by post were contacted by telephone.

### Measurement of expectation fulfilment

As previously described by Scott et al., a modified total expectation score was calculated for each time-point [10]. A pre-operative expectation score was calculated for each patient by assigning four points for each expectation graded as ‘very important’, three points for ‘somewhat important’, two points for ‘a little important’. The responses ‘I do not expect this’ and ‘this does not apply to me’ were combined to avoid ambiguity and were assigned one point. This created an overall expectation score from 18 to 72 for THA which was converted to 0–54 with 54 representing the highest level of expectations.

A postoperative fulfilment score was calculated for each patient at the early and late time-points: 4 points for expectations fulfilled”greatly”; 3 for “a lot”, 2 for “a little”; and 1 for “ I did not expect this” or “this did not apply to me”, creating an overall expectation fulfilment score ranging from 18 to 72. Again, this was converted to a fulfilment score ranging from 0 to 54, with 54 representing complete fulfilment.

### Statistical analysis

Statistical analysis was performed using Statistical Package for Social Sciences version 21.0 (SPSS Inc., Chicago, IL, USA). Once data was tested for normality, the appropriate parametric and non-parametric tests were used to assess continuous variables for significant differences between groups. An unpaired Student’s *t* test or a Mann Whitney *U* test, one-way ANOVA or a Kruskal Wallis test were used to compare linear variables between groups. The relationship between specific variables was measured using either Pearson’s (PC) or Spearman’s rank correlations (SC). Categorical variables were assessed using the Chi square test. Relative risk (RR) of dissatisfaction at late follow-up was calculated for each specific expectation question. Linear regression analysis was used to identify independent predictors of late expectation fulfilment score, entering all predictors significant (10% level or less) on univariate analysis into the model using enter methodology. A statistically significant *p* value was defined as less than 0.05.

## Results

**Fig. 1:**
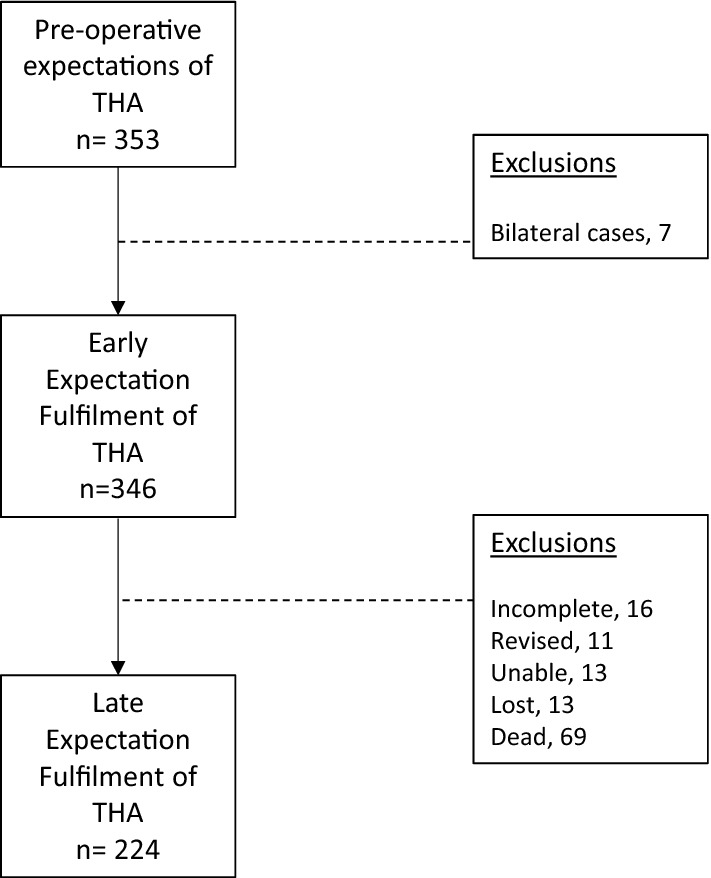
Flow diagram of study cohort

During the period of follow-up, 353 of 395 patients had complete 1-year data. Seven patients were excluded, due to undergoing bilateral procedures. At late follow-up, 69 patients had died and a further 53 patients were excluded (Fig. 1). A total of 224 patients (81% of those alive) with intact THAs with a mean follow-up of 9.5 years (range: 9.1–9.9 years) were included in this study. The social demographics, pre-operative levels of function, surgical approach and implant fixation method utilised for both responders and non-responders are summarised in Table 1. Deceased patients were older and more likely to live in deprived areas, but otherwise there were no significant differences between cohorts.

**Table 1:** Patient characteristics of long-term follow up responders and non-responders (deceased or lost)

Variable	Responders *n* = 224 (Std Dev)	Non-responders *n* = 122 (Std Dev)	*p* value
Mean age	66.9 (9.9)	71.8 (10.6)	< 0.01^a^
Female gender	137	76	0.82^c^
SIMD Quintile			
1 (Most)	5.9	7.4	0.04^c^
2	18.5	24.6	
3	17.6	18.0	
4	21.6	13.1	
5 (Least)	36.5	36.9	
Depression	12	11	0.27^c^
Pain in other joints	147	87	0.73^c^
Pre-op PROMs			
Mean OHS	19.4 (7.5)	18.4 (7.7)	0.28^b^
Mean SF-12 PCS	30.0 (6.7)	28.5 (7.2)	0.22^b^
Mean SF-12 MCS	49.2 (11.3)	49.0 (11.2)	0.60^b^
Approach			
Anterolateral	108	59	0.22^c^
Posterior	116	63	
Implant fixation			
Cemented	202	116	0.04^c^
Hybrid	8	6	
Reverse hybrid	1	0	
Uncemented	13	0	

*SIMD* Scottish Index of Multiple Deprivation; *OHS* Oxford Hip Score; *SF-12* Short Form 12; *PCS* physical component score, *MCS* mental component score

^a^Mann-Whitney *U* test

^b^Student’s *T* test

^c^Chi Squared test

### Pre-operative expectations

**Fig. 2:**
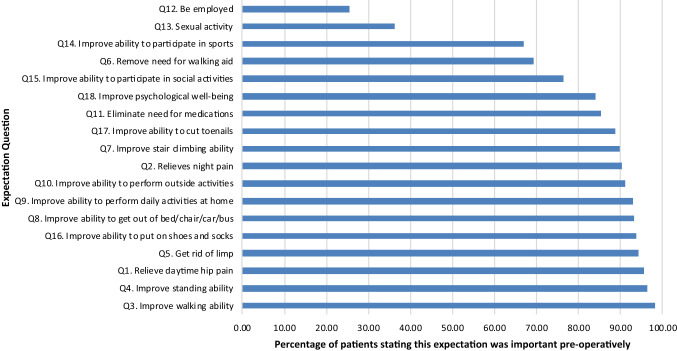
Pre-operative expectations ranked somewhat or very important in THA patients

The median preoperative expectation score was 43.5 (IQR 10) (Fig. 2). Median preoperative expectation scores were significantly higher in men [45 vs 43; difference 2.0, 95% Confidence Intervals (CI) 0.0–4.0; *p* = 0.043, Mann–Whitney *U* test]. There were no significant differences in preoperative expectation scores across SIMD quintiles (*p* = 0.099), patients with and without depression (*p* = 0.724, Mann–Whitney *U* test) or with pain in other joints (*p* = 0.427, Mann–Whitney *U* test). Higher preoperative expectation scores correlated significantly with worse preoperative PROMs: OHSs (SC − 0.358, *p* < 0.001) and PCS (SC − 0.297, *p* < 0.001).

Overall median preoperative expectation score was not associated with satisfaction at 1 year (satisfied 43.0 vs dissatisfied 45.0; 95% CI: − 6.0 to 1.0; *p* = 0.313, Mann Whitney *U* test) or at late follow-up (satisfied 43 vs dissatisfied 44.5; 95% CI: − 5.0 to 2.0; *p* = 0.886, Mann Whitney *U* Test). However, expectation fulfilment scores were significantly associated with satisfaction when measured at both early (satisfied 37.0 vs dissatisfied 19.0, difference 16.0, 95% CI: 9.0–23.0, *p* < 0.001, Mann–Whitney *U* test) and late (satisfied 35.0 vs dissatisfied 10.0; difference 21.0, 95% CI: 16.0–27.0, *p* < 0.001, Mann–Whitney *U* test) timepoints. High preoperative expectations of each specific activity did not appear to be associated with increased risk of dissatisfaction at early or long-term follow-up (Table 2). Higher preoperative expectation scores independently correlated with higher expectation fulfilment at both early and late follow-up (Table 3).

**Table 2:** Relative risk of early or late dissatisfaction in patients who ranked preoperative expectations of individual activities as very important (i.e., high expectations of that activity)

Question	*n*	Early RR (95% CI)	Late RR (95% CI)
Q1. Relieve daytime pain in the joint	200	1.01 (0.8–1.2)	1.01 (0.8–1.2)
Q2. Relieves pain in the joint that interferes with sleep	182	0.99 (0.8–1.2)	0.97 (0.8–1.2)
Q3. Improve ability to walk	212	0.94 (0.9–1.0)	1.07 (0.9–1.3)
Q4. Improve ability to stand	189	0.89 (0.8–1.0)	1.01 (0.8–1.3)
Q5. Get rid of limp	181	1.06 (0.8–1.4)	1.04 (0.8–1.3)
Q6. Remove the need for a stick or other assistive device	130	0.89 (0.6–1.3)	0.86 (0.6–1.2)
Q7. Improve ability to climb stairs	164	0.95 (0.7–1.3)	1.02 (0.8–1.4)
Q8. Improve ability to get out of bed/chair/car/bus	189	0.95 (0.8–1.1)	1.01 (0.8–1.3)
Q9. Improve ability to perform daily activities around the home	176	0.95 (0.8–1.2)	0.87 (0.7–1.0)
Q10. Improve ability to perform daily activities out of the home	162	1.03 (0.7–1.4)	0.86 (0.7–1.1)
Q11. Eliminate the need for medications	152	0.96 (0.7–1.3)	0.69 (0.6–0.8)
Q12. Be employed for monetary reimbursement	46	0.67 (0.3–1.5)	0.72 (0.3–1.6)
Q13. Sexual activity	42	1.07 (0.4–3.1)	1.75 (0.5–6.6)
Q14. Improve ability to participate in recreational sports	96	1.04 (0.6–1.9)	1.31 (0.7–2.6)
Q15. Improve ability to participate in social activities	114	0.77 (0.5–1.1)	0.91 (0.6–1.4)
Q16. Improve ability to put on shoes and socks	167	0.90 (0.7–1.1)	0.89 (0.7–1.1)
Q17. Improve ability to cut toenails	155	0.83 (0.7–1.1)	0.95 (0.7–1.3)
Q18. Improve psychological well-being	155	0.83 (0.7–1.1)	0.95 (0.7–1.3)

*RR* relative risk; *CI* confidence interval

**Table 3:** Associations of preoperative variables with expectation fulfilment at late follow-up on univariate analysis (*n* = 224)

Case mix variable	Long-Term Expectation Fulfilment Score		
	Mean ± SD	Correlation	*p* value
Age		− 0.121	0.07^a^
BMI		− 0.021	0.753^a^
Gender			
Female	41.5 ± 7.6		0.041^c^
Male	43.6 ± 7.1		
SIMD Quintile			
1	34.5 ± 13.7		0.606^b^
2	32.5 ± 13.8		
3	33.8 ± 11.7		
4	35.6 ± 11.2		
5	36.1 ± 11.9		
Depression			
Yes	34.4 ± 10.7		0.913^c^
No	34.8 ± 12.0		
Pain in other joints			
Yes	34.8 ± 11.6		0.954^c^
No	34.7 ± 12.6		
Preop expectation score		0.284	< 0.001^a^
Pre-op PROMs			
OHS		0.121	0.072^c^
SF-12 PCS		0.075	0.263^c^
SF-12 MCS		0.160	0.016^c^
Approach			
Anterolateral	31.4 ± 13.8		0.423^d^
Posterior	33.0 ± 12.8		
Implant fixation			
Cemented	32.3 ± 12.9		0.191^b^
Hybrid	23.8 ± 19.4		
Uncemented	34.6 ± 14.0		

*SD* standard deviation; *CI* confidence interval; *BMI* body mass index; *SIMD* Scottish Index of Multiple Deprivation; *OHS* Oxford Hip Score; *SF-12* Short Form 12; *PCS* physical component score, *MCS* mental component score;

^a^Pearson’s correlations

^b^ANOVA

^c^Student’s *T* test

^d^Mann-Whitney *U* test

### Late expectation fulfilment

Overall, early postoperative expectation fulfilment scores declined from 36.5 (IQR 17) to 33 (IQR 22.5) at late follow-up (difference 2.0, 95% CI 0.0–5.0, *p* < 0.001). During this time period, overall levels of satisfaction remained similar: 92% at early follow-up and 91% late (*p* = 0.359, chi-square test). Late expectation fulfilment scores were significantly associated with all other PROMs applied at both timepoints (Table 4): better outcomes were associated with greater fulfilment. Furthermore, improvements in OHS and PCS score from pre-operative baseline to late follow-up were significantly associated with late expectation fulfilment scores (Table 4). There was no significant difference in the overall number of expectations that were fulfilled only a little at early (Median 1, IQR 3) and late follow-up (Median 1, IQR 3, *p* = 0.908, Mann Whitney *U* test). There were no significant associations between expectation fulfilment scores and the surgical approach or implant fixation method used (Table 3).

**Table 4:** Associations of postoperative PROMs with late expectation fulfilment on univariate analysis (*n* = 224)

	Pearson’s Correlation	*p* value
1-year PROMs		
OHS	0.464	< 0.001
SF-12 PCS	0.384	< 0.001
SF-12 MCS	0.253	< 0.001
1-year expectation fulfilment score	0.494	< 0.001
Change from preop to 1 year		
OHS	0.246	< 0.001
SF-12 PCS	0.311	< 0.001
SF-12 MCS	0.052	0.437
Long-term PROMs		
OHS	0.685	< 0.001
SF-12 PCS	0.505	< 0.001
SF-12 MCS	0.317	< 0.001
EQ-5D	0.572	< 0.001
Change from early to long-term		
OHS	0.635	< 0.001
SF-12 PCS	0.114	0.089
SF-12 MCS	0.080	0.233
Change from pre-op to long term		
OHS	0.514	< 0.001
SF-12 PCS	0.351	< 0.001
SF-12 MCS	0.107	0.111

*OHS* Oxford HIP Score; *SF-12* Short Form 12; *PCS *physical component score, *MCS* mental component score; *EQ-5D* EuroQol-5 Dimensions questionnaire

Univariate analysis identified a number of preoperative and 1-year postoperative variables associated with long-term expectation fulfilment (Tables 3, 4). Multivariate analysis was performed to identify predictors of late expectation fulfilment following THA. Several factors were identified which independently predicted late expectation fulfilment: younger age, greater pre-operative expectation score and greater improvement in the OHS (both early and late) were all variables which added significantly to the prediction model (*p* < 0.05).

### Late fulfilment of specific expectations

**Fig. 3:**
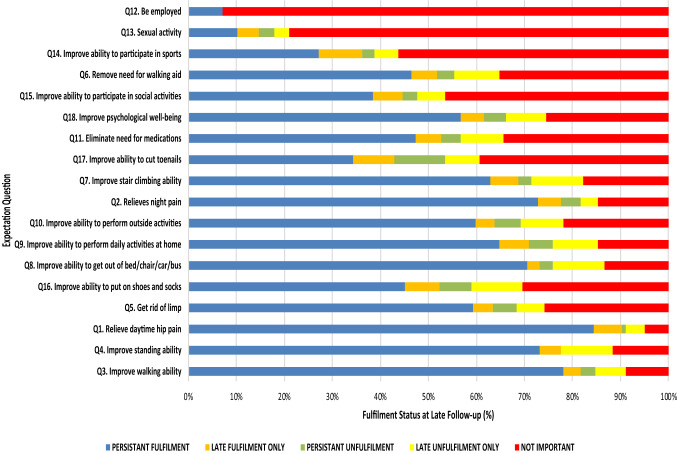
Degree of expectation fulfilment at late follow-up following THA

Late expectation fulfilment was achieved in 5–10% of patients for 17 (not including employment) of the 18 expectations assessed (Table 5). Approximately two out of every five patients who considered themselves unfulfilled at early follow-up went on to experience late fulfilment, but this was dependent upon the specific expectation (mean 40%, range: 0–64%) (Table 6). However, 5%-10% of previously fulfilled patients reported poor fulfilment at late follow-up only (Fig. 3).

**Table 5:** Predictors of expectation fulfilment at late follow-up following TKA on multivariate analysis (linear regression)

Predictors in the model (*R* = 0.746, *R*^2^ = 0.556)	*B*	95% Confidence intervals		*p* value
		Lower	Upper	
Age	− 0.223	− 0.430	− 0.17	< 0.001
Gender	0.034	− 1.670	3.552	0.478
Depression	0.084	− 0.784	11.022	0.089
Pain in other joints	0.044	− 1.531	4.032	0.376
Pre-op expectation score	0.170	0.116	0.493	0.002
Pre-op SF-12 PCS	0.083	− 0.119	0.450	0.253
1-year OHS	0.102	− 0.047	0.334	0.140
1-year SF-12 MCS	0.394	0.228	1.185	0.004
Change in OHS to 1 year	0.575	0.520	1.150	< 0.001
Change in PCS to 1 year	0.088	− 0.089	0.305	0.280
Change in MCS to 1 year	− 0.051	− 0.270	0.140	0.531
Change in OHS to 9 year	0.779	0.601	0.935	< 0.001
Change in PCS to 9 year	0.051	− 0.074	0.161	0.468
Change in MCS to 9 year	0.045	− 0.091	0.169	0.558

Pre-op OHS, pre-op SF-12 MCS and 1-year SF-12 PCS was excluded by the model

*OKS* Oxford Knee Score; *PCS* Physical Component Score

*F*(14,204) = 18.282, *p* < 0.001

**Table 6:** The likelihood of becoming fulfilled at late follow-up when unfulfilled at 1 year by activity

Pre THA importance rank	Question	*N* (%) Patients unfulfilled at early follow-up	% Patients who become Fulfilled by Late Follow-up
1	Q3. Improve ability to walk	18 (8.0)	44.4
2	Q4. Improve ability to stand	22 (9.8)	45.4
3	Q1. Relieve daytime pain in the joint	11 (4.9)	63.6
4	Q5. Get rid of limp	25 (11.2)	36.0
5	Q16. Improve ability to put on shoes and socks	43 (19.2)	37.2
6	Q8. Improve ability to get out of bed/chair/car/bus	18 (8.0)	33.3
7	Q9. Improve ability to perform daily activities around the home	36 (16.1)	38.9
8	Q10. Improve ability to perform daily activities away from home	32 (14.3)	28.1
9	Q2. Relieves pain in the joint that interferes with sleep	20 (8.9)	55.0
10	Q7. Improve ability to climb stairs	23 (10.3)	56.5
11	Q17. Improve ability to cut toenails	63 (28.1)	30.2
12	Q11. Eliminate the need for medications	31 (13.8)	38.7
13	Q18. Improve psychological well-being	29 (13.0)	37.9
14	Q15. Improve ability to participate in recreational/social activities	40 (17.9)	35.0
15	Q6. Remove the need for a stick or other assistive device	30 (13.4)	40.0
16	Q14. Improve ability to participate in recreational sports	39 (17.4)	51.3
17	Q13. Sexual activity	29 (13.0)	34.5
18	Q12. Be employed for monetary reimbursement	4 (1.8)	0.0

Fourteen out of eighteen activities demonstrated high levels of persistent expectation fulfilment at late follow-up. Relief of hip pain (daytime and at night), improved walking, the ability to stand and transfer (from the bed, chair, car or bus) were associated with high levels of persistent fulfilment (> 70%).

On average, fewer than one in twenty patients considered themselves persistently unfulfilled for specific tasks (mean 4%, range: 0–11%). Poorly met expectation fulfilment at late follow-up only was highest for ability to put on shoes and socks, sexual activity, remove need for walking aid, eliminate need for medications and improved stair climbing ability.

## Discussion

The degree of expectation fulfilment following THA changes over time for the majority of activities. Whilst overall the absolute levels of expectation fulfilment declined from early (1 year) to late (mean 9.5 years) follow-up, patients reported generally high levels of fulfilment for the majority of specific tasks measured at late follow-up. High levels of pre-operative expectations for specific activities was not associated with either early or late dissatisfaction. However, higher levels of post-operative expectation fulfilment were significantly associated with satisfaction at both time periods.

Mancuso et al. have previously reported on expectation fulfilment 4 years after THA [8]. These authors transformed the total expectations score to a number between 0 and 100 and considered a change in the same direction for five expectations as an important clinical change, corresponding to a change in 6 points in a transformed score. Their study identified that better pre-operative and 4-year post-operative PROMs scores were closely associated with the fulfilment of expectations following THA. The current study demonstrates that these findings continue up to 10 years following surgery. In addition, fulfilment of patient expectations can still be achieved after 1 year following THA, with approximately 40% of poorly-fulfilled patients reporting improvements at late follow-up.

A previous qualitative study has demonstrated variability in patients' pre-operative expectations of THA [9]. The results of the current study complement these findings and confirm that variation occurs in each subject’s consideration of whether a certain expectation was, or was not, applicable to them.

Younger age at time of surgery was shown to be an independent predictor of overall late expectation fulfilment on multivariate analysis. This corresponds with previous studies which have demonstrated an association between younger age at surgery and higher levels of expectation fulfilment, albeit at earlier time points [8, 10].

It has been suggested that pre-operative expectations of total joint arthroplasty can be modified by pre-operative education classes, thus leading to greater post-operative fulfilment and satisfaction [8, 10, 19]. However, a previous randomised controlled study demonstrated no significant differences in the ‘within-patient change’ of expectation fulfilment scores when receiving educational classes vs standard information provided in the pre-assessment clinic (e.g., information booklet) [20]. The current study results have demonstrated that there is variation in what is felt to be important to individual patients, and therefore, pre-operative discussions of outcome should be tailored to each patient’s needs and life-goals. Furthermore, pre-operative function appears to be an independent predictor of expectation fulfilment. Though not examined in the current study, previous studies have suggested that delaying surgery may be detrimental to the subsequent outcome achieved [8, 21].

The main limitations of this study include the loss to follow-up with 69 patients (19.9%) deceased and a further 53 patients (15.3%) excluded at late follow-up (Fig. 1). Comparison between the ‘responder’ and ‘non-responder’ groups demonstrated that non-responder patients were older and more likely to live in deprived areas. This is to be expected as the majority of non-responders were either deceased or unable to complete the questionnaire due to dementia.

There was an all-cause revision rate of 3.1% (*n* = 11) at 10 years following primary THA surgery. Given that the aim of this study was to determine how expectation fulfilment scores changed over time following primary THA, we deliberately chose to exclude patients who had underwent revision surgery as it would skew the overall results and may not accurately represent those patients with intact THAs at 10 years. Therefore, the results of this study should be used to counsel patients assuming that they might not require revision surgery within 10 years.

The current study did not measure the effect of race or underlying diagnosis. In addition, a further clinical assessment of each patient was not performed, which may be important as lower expectation fulfilment has previously been associated with limp and/or leg length discrepancy [8]. However, objective and validated measures of hip joint performance (OHS) were employed as a quantification of overall function.

The current cohort appears representative of a ‘typical’ arthroplasty cohort –expectation fulfilment in the extremes of age may not be accurately reflected in our results and only a small proportion of patients were young (i.e., below 55 years). It is possible that pre-operative expectations and their fulfilment in young working age patients may differ compared to the more typical older THA population. More work is required to establish pre-operative expectations and their subsequent fulfilment in young, working-age patients undergoing THA.

Finally, we did not perform a pre-study power calculation. Although previous authors have sought to use the ‘within-patient change’ as a marker of successful expectation fulfilment [20], there is no documented mean clinically important difference of the HSS hip expectations score. Therefore, the clinical relevance of statistical significance is uncertain, and it is difficult to adequately determine the necessary power required to measure change in expectation fulfilment score.

## Conclusions

The degree of expectation fulfilment for specific activities following primary THA at late follow-up remains high. High levels of pre-operative expectations was not associated with dissatisfaction at either early or late follow-up. However, higher levels of post-operative expectation fulfilment were significantly associated with satisfaction at both time periods. Where patients considered themselves unfulfilled for a specific activity at early follow-up, on average two out of every five would go on to experience later fulfilment. This information can be used to help manage expectations in to the longer term of patients undergoing THA, particularly those who may report early unfulfillment with specific tasks.
